# Plasma d-asparagine and the d/l-serine ratio reflect chronic kidney diseases in children regardless of physique

**DOI:** 10.1007/s00726-024-03400-x

**Published:** 2024-06-06

**Authors:** Toshimasa Morishita, Naoto Nishizaki, Sakiko Taniguchi, Shinsuke Sakai, Tomonori Kimura, Masashi Mita, Mayu Nakagawa, Amane Endo, Yoshiyuki Ohtomo, Masato Yasui, Toshiaki Shimizu, Jumpei Sasabe

**Affiliations:** 1 Department of Pharmacology, Keio University School of Medicine, 35 Shinanomachi, Shinjuku-ku, Tokyo, 160-8582 Japan; 2 Department of Pediatrics and Adolescent Medicine, Juntendo University Graduate School of Medicine, Tokyo, Japan; 3 Department of Pediatrics, Juntendo University Urayasu Hospital, Chiba, Japan; 4 Department of Nephrology, Osaka University School of Medicine, Osaka, Japan; 5 KAGAMI Inc, Osaka, Japan; 6 Department of Pediatrics, Juntendo University Nerima Hospital, Tokyo, Japan; 7 Human Biology-Microbiome-Quantum Research Center (WPI-Bio2Q), Keio University, Tokyo, Japan

**Keywords:** Juvenile kidney function, d-amino acids, Body size, Clinical laboratory test

## Abstract

Biomarkers that accurately reflect renal function are essential in management of chronic kidney diseases (CKD). However, in children, age/physique and medication often alter established renal biomarkers. We studied whether amino acid enantiomers in body fluids correlate with renal function and whether they are influenced by physique or steroid medication during development. We conducted a prospective study of children 2 to 18 years old with and without CKD. We analyzed associations of serine/asparagine enantiomers in body fluids with major biochemical parameters as well as physique. To study consequences of kidney dysfunction and steroids on serine/asparagine enantiomers, we generated juvenile mice with uninephrectomy, ischemic reperfusion injury, or dexamethasone treatment. We obtained samples from 27 children, of which 12 had CKD due to congenital (*n* = 7) and perinatal (*n* = 5) causes. Plasma d-asparagine and the d/l-serine ratio had robust, positive linear associations with serum creatinine and cystatin C, and detected CKD with high sensitivity and specificity, uninfluenced by body size or biochemical parameters. In the animal study, kidney dysfunction increased plasma d-asparagine and the d/l-serine ratio, but dexamethasone treatment did not. Thus, plasma d-asparagine and the d/l-serine ratio can be useful markers for renal function in children.

**Supplementary Information:**

The online version contains supplementary material available at 10.1007/s00726-024-03400-x.

## Introduction

Chronic kidney disease (CKD) is a state of structural or functional kidney deterioration that persists for more than three months and progresses gradually to end-stage renal disease. While adult CKD is often acquired due to lifestyle-related diseases, most cases of pediatric CKD have congenital/perinatal origins. End-stage renal failure due to CKD results in the need for renal transplantation, or reduced quality of life, cardiovascular problems, and early death (Levey et al. 2005). Therefore, early detection and treatment of pediatric CKD are essential.

Over the past two decades, the occurrence of CKD in children has steadily increased (Saran et al. 2015). Among the causes of pediatric CKD, congenital anomalies of the kidney and urinary tract (CAKUT) and hereditary nephropathies account for approximately two thirds of all cases in developed countries, whereas acquired causes predominate in developing countries (Harambat et al. 2012). CAKUT includes hypoplastic kidneys, dysplastic kidneys, renal aplasia, obstructive urinary tract disease due to posterior urethral valves, and various lower urinary tract disorders. A large cohort study of children with CAKUT described the natural history of renal function and reported that about 40–50% of cases showed a gradual decline in renal function from 3 years of age until the end of puberty (Gonzalez Celedon et al. 2007). On the other hand, an increasing cause of pediatric CKD in recent years is low birthweight (LBW) (Reidy and Rosenblum 2009). The prevalence of LBW is high in developing countries and increasing in most developed countries, which will increase the incidence of CKD associated with prematurity (Luyckx and Brenner 2015; Nishizaki and Shimizu 2022). In preterm and/or LBW infants, nephrogenesis continues after birth for several weeks, but these infants ultimately have fewer functional nephrons (Sutherland et al. 2011). Therefore, incomplete nephrogenesis due to unfavorable perinatal circumstances are now regarded as a risk factor for CKD later in life.

Classically, the gold standard for assessing renal function, glomerular filtration rate (GFR), is measurement of inulin clearance in both immature and mature kidneys (Iacobelli and Guignard 2021). Inulin is freely filtered by the glomerulus, is not secreted or reabsorbed in the tubules, and is not synthesized or metabolized by the kidneys. Whereas inulin has properties of an ideal kidney marker, measurement of inulin clearance requires a constant intravenous infusion of inulin and timed collection of plasma and urinary specimens. Also, urinary catheterization is often required in children; therefore, measurement of inulin clearance is not practical for children. As an alternative, plasma disappearance techniques following a single injection of exogenous markers, such as inulin or iohexol, can be used to evaluate GFR (van Rossum et al. 2005; Schwartz et al. 2006).

On the other hand, in clinical practice, endogenous serum markers, including creatinine, cystatin C (CysC), urea, β-2 microglobulin (β2MG), and β-trace protein, are widely used to estimate GFR in children (den Bakker et al. 2018). Currently, serum creatinine is the most commonly used marker, but it varies with age and muscle mass (Stevens et al. 2006). The level is high at birth, reflecting maternal creatinine, decreases rapidly to a neonatal level in 1 or 2 weeks, and then gradually increases until 15 years of age (Kuhlback et al. 1968). Unlike creatinine, CysC and β2MG do not cross the placental barrier and can be used to assess kidney function of newborns (Cataldi et al. 1999; Bokenkamp et al. 2001). CysC can be more accurate than creatinine among children in which muscle mass is altered, as in cases of malignant diseases (Blufpand et al. 2011). As CysC is less sensitive to age and muscle mass than creatinine, CysC can serve as a suitable endogenous marker of renal function in children (Roos et al. 2007; Ferguson et al. 2015). However, CysC is rarely excreted in the urine, and its behavior differs from that of inulin. In addition, CysC is affected by hyperthyroidism (Fricker et al. 2003), obesity (Knight et al. 2004), febrile illness (Randers et al. 1998), and intake of glucocorticoids (Risch et al. 2001, 2005; Bokenkamp et al. 2007). Therefore, in addition to existing renal markers, other endogenous markers that represent renal function are needed in pediatric practice.

Kidneys are crucial in maintaining appropriate levels of amino acid enantiomers (Sasabe et al. 2014; Kimura et al. 2016; Hesaka et al. 2019; Gonda et al. 2023). Amino acids are freely filtered by renal glomeruli, but are reabsorbed differently, depending on their chirality. Whereas 95–99% of l-enantiomers are reabsorbed in the tubules (Silbernagl et al. 1975; Young and Freedman 1971), d-enantiomers are reabsorbed only in part (Silbernagl et al. 1999) by transporters such as sodium-coupled monocarboxylate transporters (SCMTs) (Wiriyasermkul et al. 2024), and degraded enantio-selectively by d-amino acid oxidase in the tubules (Krebs Biochem J 1935). Although knowledge of d-amino acid dynamics in the kidneys is limited mostly to d-serine, accumulating evidence suggests that several d-amino acids in the blood are associated with renal function. In human healthy adults, blood d-amino acids, such as d-serine, d-alanine, d-proline, and d-asparagine, are positively correlated with creatinine and CysC (Suzuki et al. 2022). A seminal study in adult CKD shows that blood d-amino acids are associated with earlier progression to end-stage renal disease (Kimura et al. 2016). Among detectable d-amino acids, d-serine and d-asparagine correlate well with GFR measured by inulin clearance (Hesaka et al. 2019; Taniguchi et al. 2023). However, whether d-amino acids also reflect kidney function in children remains uncertain. In this study, we recruited 27 children with CKD or non-CKD and examined correlations between blood d-amino acids and renal biomarkers.

## Materials and methods

### Human samples

This study was approved by the institutional Ethics Committee and conducted in accordance with the Helsinki Declaration and institutional guidelines for clinical studies at Juntendo University Hospital. We obtained consent from parents of 27 patients (2–18 years of age), who visited the pediatric nephrology outpatient clinics of Juntendo University Hospital, Juntendo University Nerima Hospital, Juntendo University Urayasu Hospital, and Juntendo University Shizuoka Hospital between July 2022 and April 2023.

Patients with immune-mediated renal diseases, congenital heart diseases, hematologic oncologic diseases, or thyroid diseases were excluded. Those with creatinine-based estimated GFR (eGFR_Cre) < 90 mL/min/1.73 m^2^ were classified as having CKD, and those with eGFR_Cre ≥ 90 mL/min/1.73 m^2^ were enrolled in the control group (Uemura et al. 2014a).

The control group included individuals with primary enuresis or false-positive urinalysis. Plasma and urine samples were collected from all subjects as a routine laboratory test during outpatient visits. Clinical information such as sex, perinatal history, and disease status of patients was obtained from medical records.

### Sample collection

Blood samples were drawn from the brachial vein, collected in Na_2_EDTA collection tubes (TERUMO, Tokyo, Japan), and stored at 4 ˚C for 3–6 h before centrifugation. After centrifugation at 1200 x *g* for 10 min, plasma was isolated and stored at -80 ˚C until analysis. Spot urine was stored at -80 ˚C until analysis.

### Animals

All animal experiments were approved by the institutional Animal Experiment Committee and conducted in accordance with Institutional Guidelines on Animal Experimentation at Keio University. C57BL/6J mice were purchased from CLEA Japan (Tokyo, Japan) and housed in a specific pathogen-free environment. Mice had free access to food and water in a light-controlled room with a 12-h light/dark cycle.

### Uninephrectomized and renal IRI model

C57BL/6J male mice at 6 weeks of age underwent procedures for uninephrectomy and ischemia-reperfusion injury (IRI), as previously reported (Sasabe et al. 2014), with minor modifications. Briefly, under continuous inhalation of isoflurane, a small flank incision was made and the right kidney was removed. After 7 days, these mice were randomly assigned to two groups: a uninephrectomized group and an IRI group. Mice were anesthetized with isoflurane, and a small flank incision was made to expose the left kidney.

Blood flow in the left renal artery and vein was interrupted with a non-traumatic clamp (Schwartz Micro Serrefines; Fine Science Tools Inc., Vancouver, Canada), and the clamp was removed after 45 min of ischemia. The abdomen was closed after visual confirmation that the surface color of the kidney had returned to normal. Sham-operated control mice were treated in the same manner except for removal of the right kidney and left kidney ischemia. At 24 h after reperfusion, under isoflurane, blood and urine were collected from the inferior vena cava and bladder, respectively.

### Dexamethasone-treated model

At 6 weeks of age, C57BL/6J male mice received intraperitoneal administration of dexamethasone (Dex, 20 mg/kg) or vehicle (saline) at 24-h intervals for 3 consecutive days. Blood and urine were collected from the inferior vena cava and bladder, respectively, under isoflurane, 24 h after the last Dex administration.

### Quantification of chiral amino acids

d- and l-amino acids in body fluids were quantified using a two-dimensional high-performance liquid chromatography (2D-HPLC) system (NANOSPACE SI-2 series, Shiseido, Tokyo, Japan), as previously described. Briefly, samples were de-proteinated with 19.5 volumes of methanol. The mixture was vortexed vigorously and centrifuged at 12,100 × *g* for 5 min at 4 °C. Supernatant was evaporated to dryness, suspended in 200 mM sodium borate, and then derivatized with 4-fluoro-7-nitro-2,1,3-benzoxadiazole (NBD-F). NBD-conjugated amino acids were separated on an octadecylsilyl (ODS) column (Singularity RP-18, 1.0 mm inner diameter (ID) x 250 mm) (designed by Kyushu University and KAGAMI Co. Ltd., Osaka, Japan) for 1D separation.

Enantiomers of amino acids were separated using a Pirkle-type enantioselective column (Singularity CSP-001 S, 1.5 mm ID x 250 mm) for 2D separation (designed by Kyushu University and KAGAMI). Fluorescence of NBD-amino acids was detected at 530 nm with excitation at 470 nm.

### Statistical analysis

No statistical methods were used to predetermine sample size. Statistical significance was determined with two-tailed, unpaired t-tests to compare two groups. *P* < 0.05 was considered significant. Simple correlation was analyzed using Spearman’s correlation coefficient in R studio (https://github.com/rstudio/) and visualized using the corrplot package (https://CRAN.R-project.org/package=corrplot). Simple linear regression was analyzed to study the relationship between two groups of variables. Data plotting and statistical analyses were performed using Prism 9.3.0 (GraphPad Software, La Jolla, CA, USA).

## Results

### Characteristics of human subjects

During the study period, we examined 27 subjects (13 males and 14 females). Of those, 12 patients were diagnosed as having CKD (CKD group), and the remaining 15 subjects comprised the control group. Characteristics of subjects are summarized in Table 1. Primary causes of CKD were perinatal (preterm, *n* = 5) or congenital (multicystic dysplastic kidney, *n* = 3; vesicoureteral reflux, *n* = 2; bilateral hypoplastic kidney, *n* = 1; and bilateral ureteropelvic junction obstruction, *n* = 1). Controls were subjects with primary enuresis (*n* = 8), false positive urine tests (*n* = 5), and frequent urination (*n* = 2). The control group was selected from patients who were negative for proteinuria, hematuria, leukocyturia, and urinary sugar on urinalysis at the time of their visits to the hospital. In the CKD group, serum creatinine, CysC, and β2MG were significantly higher than in controls, and eGFR_Cre and CysC-based eGFR (eGFR_CysC) were lower than 90 mL/min/1.73m^2^, meeting CKD criteria (Table 1) (Uemura et al. 2014b; Ikezumi et al. 2015). The CKD group had a significantly higher proportion of males than the control group. Although there were no statistical differences between the CKD and control groups in age, body size, birth weight, or biochemical non-renal parameters, we observed trends of higher age and body weight in the CKD group (Table 1 and Supplementary Table 1). These trends may have occurred since CKD subjects were recruited from patients undergoing clinical follow-up for years after the initial diagnosis. On the other hand, among CKD patients, body size, age, and sex in patients with perinatal causes (preterm group) did not differ from those in patients with congenital causes (CAKUT group) (Supplementary Table 2).

**Table 1:** Characteristics of CKD and control group

	CKD (*n* = 12)	Control (*n* = 15)	*P* value
Age, y	12.3 ± 5.4	6.5 ± 2.8	0.12
Male (%)	10 (83)	3 (20)	0.0018 **
Height, cm	149.1 ± 30.1	116.6 ± 14.2	0.22
Body weight, kg	44.3 ± 22.2	19.7 ± 7.4	0.17
Body mass index, kg/m²	17.9 ± 4.8	15.8 ± 2.1	0.37
Body surface area, m^2^	1.39 ± 0.48	0.79 ± 0.18	0.083
Gestational age, week	38.0 ± 5.7	39.0 ± 1.8	0.47
Birth weight, g	2923 ± 1102	3036 ± 577	0.37
Creatinine in the serum, mg/dL	0.81 ± 0.36	0.35 ± 0.071	< 0.0001 ****
Cystatin C in the serum, mg/dL	1.17 ± 0.39	0.80 ± 0.10	< 0.0001 ****
β2-microglobulin in the serum, mg/dL	2.4 ± 0.99	1.3 ± 0.34	0.0001 ***
d-asparagine in the plasma, nmol/mL	0.187 ± 0.080	0.118 ± 0.027	0.0002 ***
l-asparagine in the plasma, nmol/mL	49.8 ± 8.6	45.1 ± 9.6	0.24
d-/l-asparagine ratio in the plasma, %	0.412 ± 0.11	0.278 ± 0.07	0.0006 ***
d-serine in the plasma, nmol/mL	2.29 ± 0.70	1.84 ± 0.31	0.06
l-serine in the plasma, nmol/mL	113 ± 15.0	142 ± 19.2	0.0004 ***
d-/l-serine ratio in the plasma, %	2.21 ± 0.70	1.35 ± 0.16	< 0.0001 ****
Urinary d-asparagine, nmol/mgCre	22.1 ± 13.4	31.2 ± 37.4	0.21
Urinary l-asparagine, nmol/mgCre	74.1 ± 55.3	131.9 ± 71.3	0.032 *
Urinary d-serine, nmol/mgCre	138.9 ± 52.5	271.2 ± 0.46.5	< 0.0001 ****
Urinary l-serine, nmol/mgCre	163.2 ± 147.1	325.5 ± 156.7	0.028 *
d-/l-asparagine ratio in the urine, %	23.5 ± 16.3	20.4 ± 7.1	0.24
d-/l-serine ratio in the urine, %	104.6 ± 75.5	84.9 ± 30.2	0.32
Creatinine based FE_D−asparagine_, %	67.2 ± 26.2	68.1 ± 14.8	0.58
Creatinine based FE_D−serine_, %	45.5 ± 11.4	48.5 ± 7.3	0.34
eGFR_Cre, mL/min/1.73 m²	77.0 ± 19.0	116.5 ± 12.8	< 0.0001 ****
eGFR_CysC, mL/min/1.73 m²	81.2 ± 21.1	122.3 ± 15.7	< 0.0001 ****
eGFR_β2-microglobulin, mL/min/1.73 m²	71.7 ± 25.4	115.6 ± 26.8	0.0002

### Plasma d-amino acids correlate with established biomarkers for renal function

**Fig. 1:**
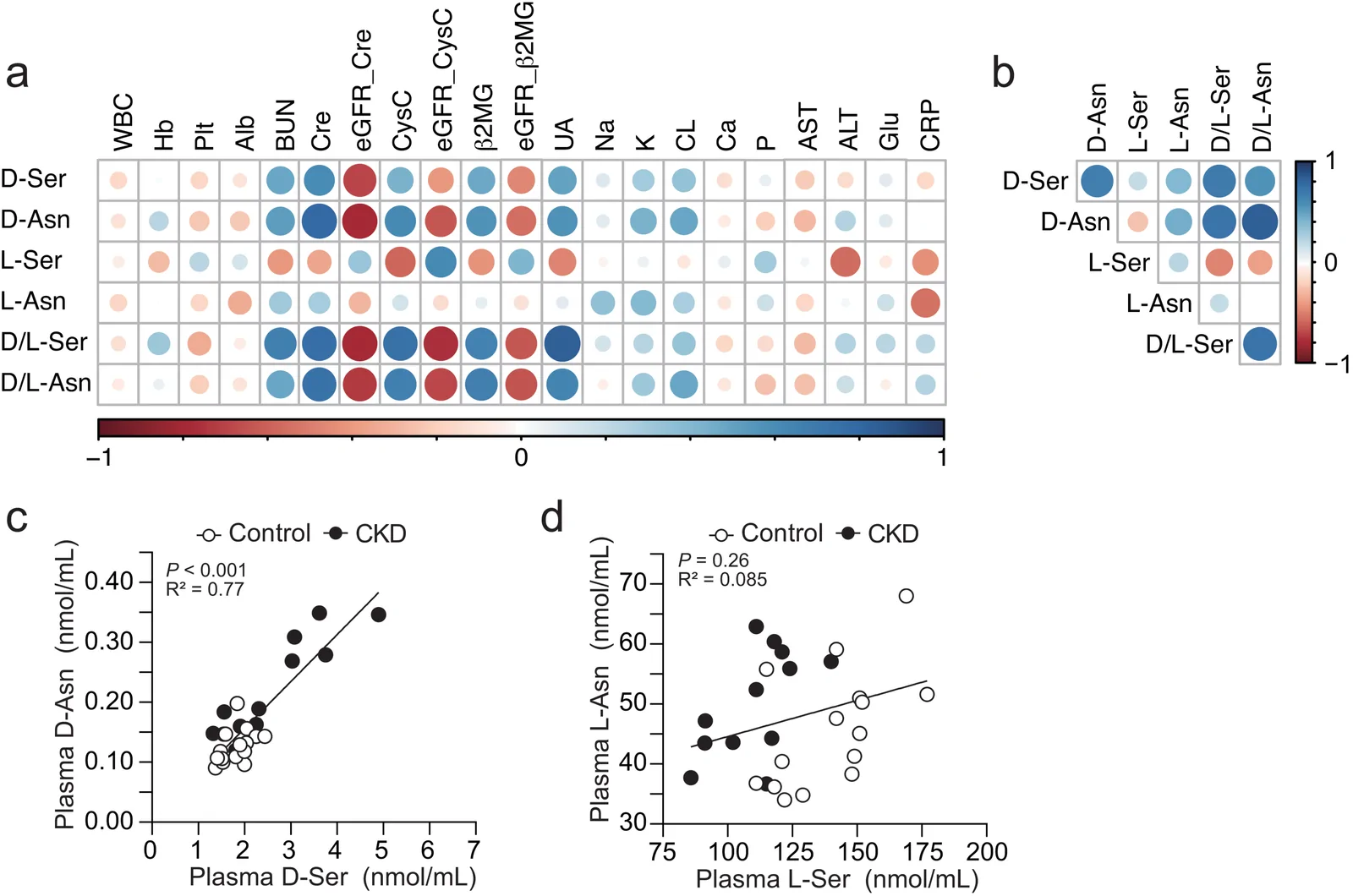
Correlations of plasma amino acid enantiomers with biochemical parameters in children with CKD and controls. (**a, b**) Heatmaps indicate Spearman’s correlation coefficient between amino acid enantiomers and major biochemical parameters (**a**), or between amino acid enantiomers (**b**) in plasma of children with CKD and controls. WBC, white blood cells; Hb, hemoglobin; Plt, platelets; Alb, albumin; BUN, blood urea nitrogen; Cre, creatinine; UA, uric acid; Glu, glucose. (**c, d**) Linear regressions between d-asparagine and d-serine (**c**) or between l-asparagine and l-serine (**d**) in the plasma are shown. Closed circles, CKD (*n* = 12); open, Control (*n* = 15)

Among detectable d-amino acids in blood, d-serine and d-asparagine have clear negative associations with inulin-based GFR in adults (Hesaka et al. 2019; Taniguchi et al. 2023). To measure d-serine and d-asparagine in pediatric samples, we used a two-dimensional HPLC system to quantify enantiomers of amino acids enantio-selectively, as previously reported (Ishii et al. 2018). Overall, plasma d-serine, d-asparagine, d/l-serine ratio, and d/l-asparagine ratio showed marked positive correlations with renal biomarkers, such as serum blood urea nitrogen (BUN), creatinine, CysC, and b2MG, whereas they correlated negatively with eGFR_Cre, eGFR_CysC, and eGFR_ β2MG in the child population (Fig. 1a). Also, as uric acid (UA) is excreted into urine, UA level paralleled plasma d-serine and d-asparagine as well as renal biomarkers (Fig. 1a). On the other hand, plasma l-serine had moderate opposite associations with renal markers from those of plasma d-serine or d-asparagine (Fig. 1a). In accordance with our previous observations in adults (Suzuki et al. 2022), d-serine and d-asparagine did not correlate with non-renal biochemical parameters in children (Fig. 1a). Plasma d-serine had positive linear associations with d-asparagine and the d/l-asparagine ratio, and plasma d-asparagine correlated with d-serine and the d/l-serine ratio (Fig. 1bc), suggesting that dynamics of d-serine and d-asparagine are similar. In contrast, plasma l-serine and l-asparagine did not show such associations (Fig. 1bd).

### Plasma d-asparagine and d/l-serine ratio can detect CKD with high specificity and sensitivity

**Fig. 2:**
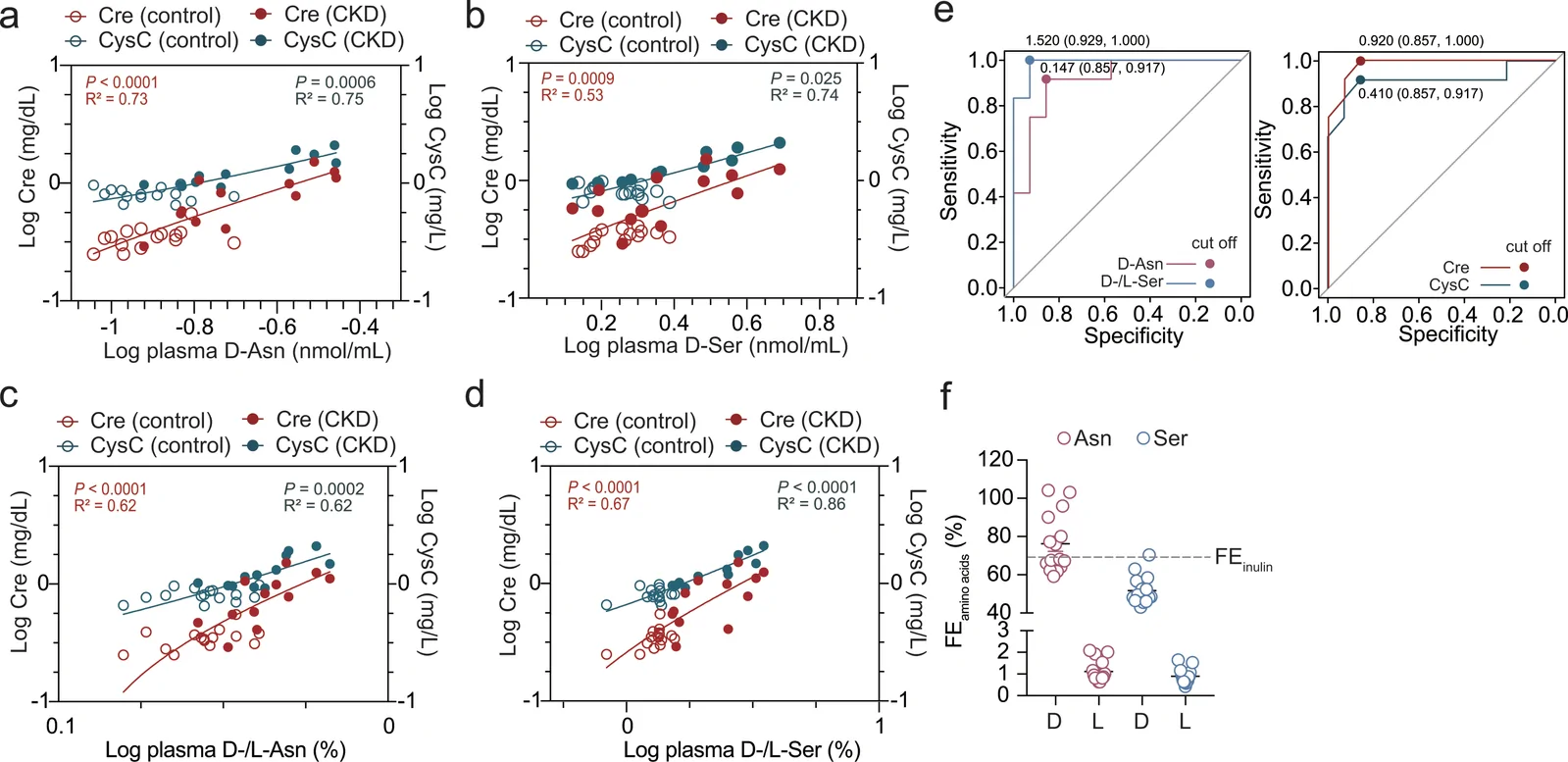
Plasma d-amino acids and d/l-amino acid ratios correlate with existing renal markers and can detect pediatric CKD. (**a-d**) Simple linear regression shows associations of plasma d-asparagine (a), d-serine (b), d/l-asparagine (c), and d/l-serine (d) with serum Cre and CysC in children with CKD (closed circles, *n* = 12) and controls (open circles, *n* = 15). (**e**) The true/false positive rates to detect pediatric CKD at each threshold setting for d-asparagine (left), d/l-serine ratio (left), creatinine (right), and CysC (right) are plotted. Values in the plots show cut offs (specificity, sensitivity). (**f**) FEs of amino acids based on Cre clearance are shown. The dotted line is the FE of inulin, based on Cre in adults

The CKD group, regardless of its causes, had significantly higher plasma d-asparagine and d/l-asparagine ratios than the control group (Table 1). Of note, plasma d-asparagine showed clear linear associations with creatinine and CysC (Fig. 2a). In the CKD group, in accordance with tendencies of higher creatinine and CysC in patients with CAKUT than those with perinatal causes, d-asparagine was significantly elevated in patients with CAKUT compared to those with perinatal causes (Supplementary Table 2). In a similar manner to d-asparagine, plasma d-serine had a tendency to be increased in the CKD group, albeit non-significantly (Table 1), and had positive linear associations with creatinine and CysC (Fig. 2b). On the other hand, plasma l-asparagine was not affected by CKD and had no correlation with creatinine or CysC (Table 1 and Supplementary Fig. 1a). Plasma l-serine in the CKD group was significantly lower than that in controls and showed negative associations with CysC (Table 1; Fig. 1b), resulting in higher d/l-serine ratios in the CKD group (Table 1). d/l-ratios of both asparagine and serine had statistically robust linear associations with creatinine and CysC (Fig. 2cd). We further studied which of those amino acid parameters in plasma can detect pediatric CKD accurately. Receiver operating characteristic (ROC) curves showed that d-asparagine and the d/l-serine ratio exhibit high sensitivity and specificity over 0.8 with cut-off values of 0.147 nmol/mL (d-asparagine) and 1.520% (d/l-serine ratio) in the tested population, which was comparable to existing biomarkers such as creatinine (cutoff, 0.920 mg/dL) and CysC (cutoff, 0.410 mg/L) (Fig. 2e).

### d-asparagine shows fractional excretion similar to that of inulin in children

To examine the pertinence of amino acid enantiomers as biomarkers for renal function, we calculated fractional excretion of the amino acids (FE_amino acids_). As FE is defined as the percentage of a filtered molecule that is excreted in the urine, high FE indicates either low reabsorption by renal tubules or substrate secretion by them. Practically, FE is calculated as the ratio of the clearance of the molecule in question to creatinine clearance. Among tested amino acids, FE_d−Asn_ showed the highest value (68%) on average, which is comparable to FE_inulin_ based on creatinine clearance (Taniguchi et al. 2023), followed by FE_d−Ser_ (Fig. 2f; Table 2). In contrast, FEs of l-amino acids were far lower than those of d-amino acids (Fig. 2f), reflecting highly efficient reabsorption of l-amino acids in renal tubules. Since creatinine is not reabsorbed, but is secreted from the tubules (Carrie et al. 1980), FE based on creatinine is thought to underestimate FE_d−Asn_ and FE_inulin_, which is believed to be nearly 100%. Therefore, our results suggest that d-asparagine has similar dynamics to those of inulin, and represents renal function in the pediatric population.

**Table 2:** FE of creatinine, d-asparagine, and d-serine based on creatinine or d-asparagine

Substrate for FE	Creatinine based FE	d-asparagine based FE
Creatinine	100.00 (Ref)	146.9 (114.4–154.9)
d-asparagine	68.1 (64.6–87.6)	100.00 (Ref)
d-serine	48.3 (45.9–55.7)	67.9 (61.3–76.7)

### Urinary d-serine roughly predicts eGFR

**Fig. 3:**
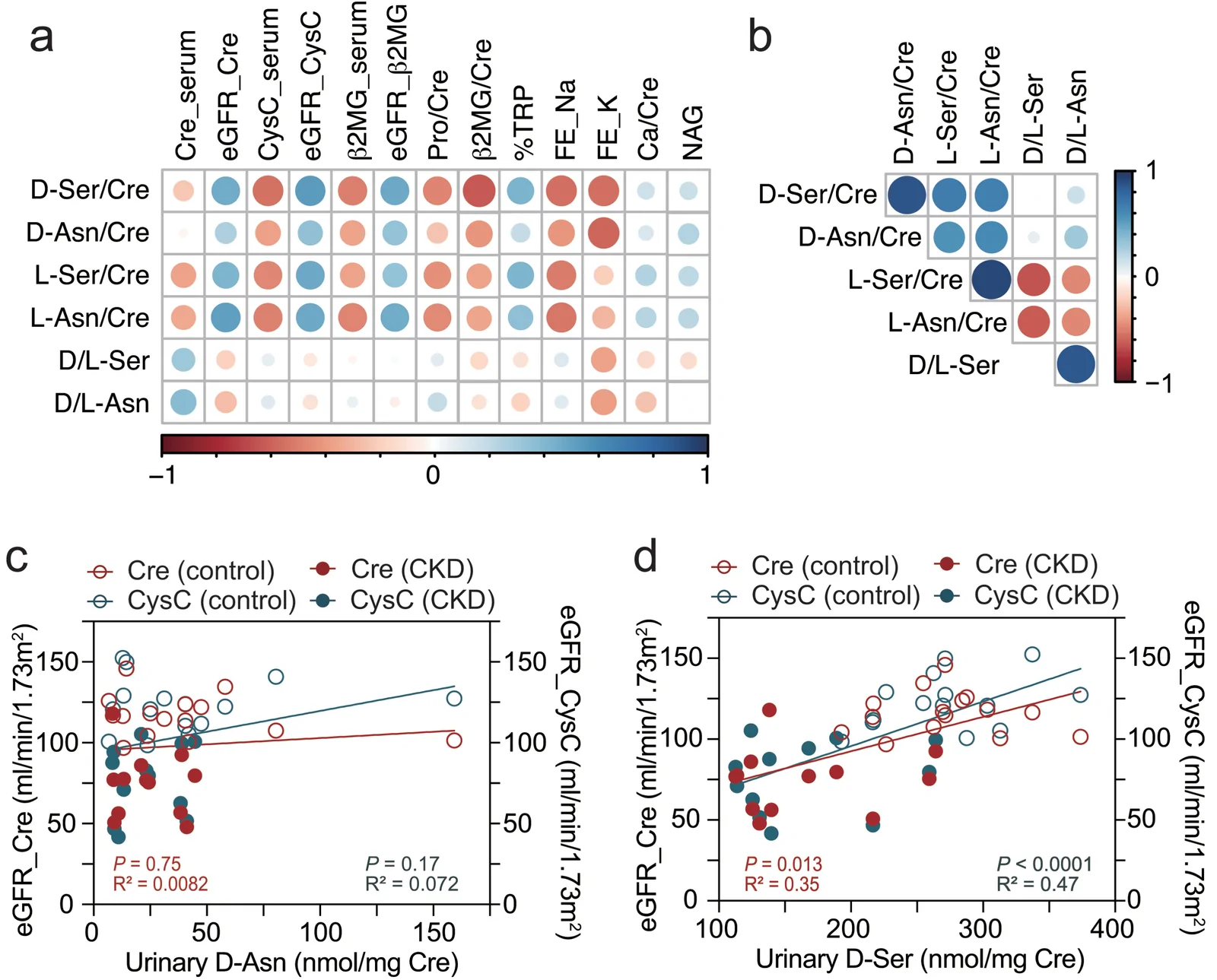
Correlations of urinary amino acid enantiomers with kidney markers in children with CKD, and controls. (**a, b**) Heatmaps indicate Spearman’s correlation coefficient between amino acid enantiomers and major kidney parameters (**a**), or between amino acid enantiomers (**b**) in urine of children with CKD and in controls. Renal parameters are urinary, unless noted as ‘serum.’ NAG, N-acetyl-beta-d-glucosaminidse. (**c, d**) Simple linear regression shows associations of urinary d-asparagine (**c**) or d-serine (**d**) with eGFR_Cre and eGFR_CysC in children with CKD (closed circles, *n* = 12) and in controls (open circles, *n* = 15)

We further tested whether urinary asparagine or serine enantiomers are associated with renal function. Urinary amino acid concentrations were standardized by those of urinary creatinine. All tested serine and asparagine enantiomers had similar, moderate positive correlations with eGFR_Cre, eGFR_CysC, and eGFR_β2MG (Fig. 3a). In contrast to the pattern in blood, not only d-serine and d-asparagine, but their l-counterparts had positive correlations (Fig. 3b and Supplementary Fig. 2ab). As a result, d/l-serine and d/l-asparagine ratios in urine did not show significant associations with established renal markers (Fig. 3a). Among serine and asparagine enantiomers in the urine, urinary d-serine showed the strongest associations with blood renal biomarkers (Fig. 3cd and Supplementary Fig. 2cd) as well as with urinary β2MG and % tubular reabsorption of phosphate (%TRP), indicating that urinary d-serine reflects tubular function (Fig. 3a).

### Plasma d-asparagine and urinary d-serine are not affected by body size or age

**Fig. 4:**
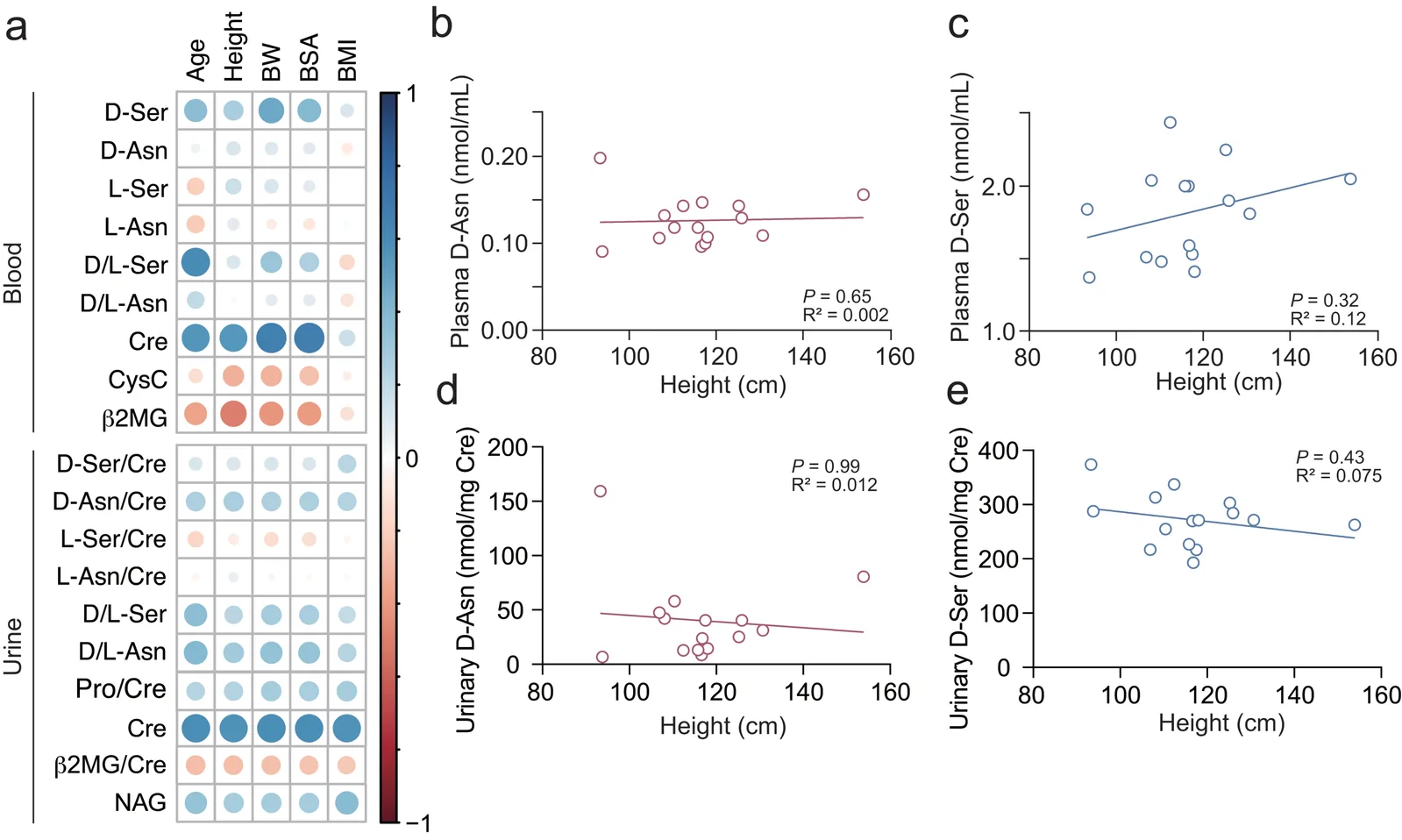
Correlations of plasma/urinary amino acid enantiomers with age or body size in non-CKD children. (**a**) Heatmap shows Spearman’s correlation coefficient between plasma/urinary amino acid enantiomers or existing kidney parameters and age / body size in control children (*n* = 15). BW, body weight; BSA, body surface area; BMI, body mass index. (**b**-**e**) Simple linear regression shows associations of plasma/urinary d-asparagine (**b**, **d**) or d-serine (**c**, **e**) with heights of control children (*n* = 15)

During development, biochemical markers that correlate with body size and age are not ideal for use as indicators of organ functions. We next tested whether plasma or urinary d-amino acids correlate with age or body size in non-CDK controls. Whereas serum and urinary creatinine had clear positive associations with age, height, body weight, and body surface area (BSA) (Fig. 4a and Supplementary Fig. 3ab), serum CysC, plasma d-asparagine, or urinary d-serine had no associations with them (Fig. 4a-e and Supplementary Fig. 3c-h). On the other hand, although plasma d/l-serine ratio was strongly correlated with established renal markers (Fig. 1a and Supplementary Fig. 1b), it was positively associated with age (Fig. 4a).

### In mice, renal dysfunction causes increased plasma d-asparagine and d/l-serine ratios, but glucocorticoids do not

**Fig. 5:**
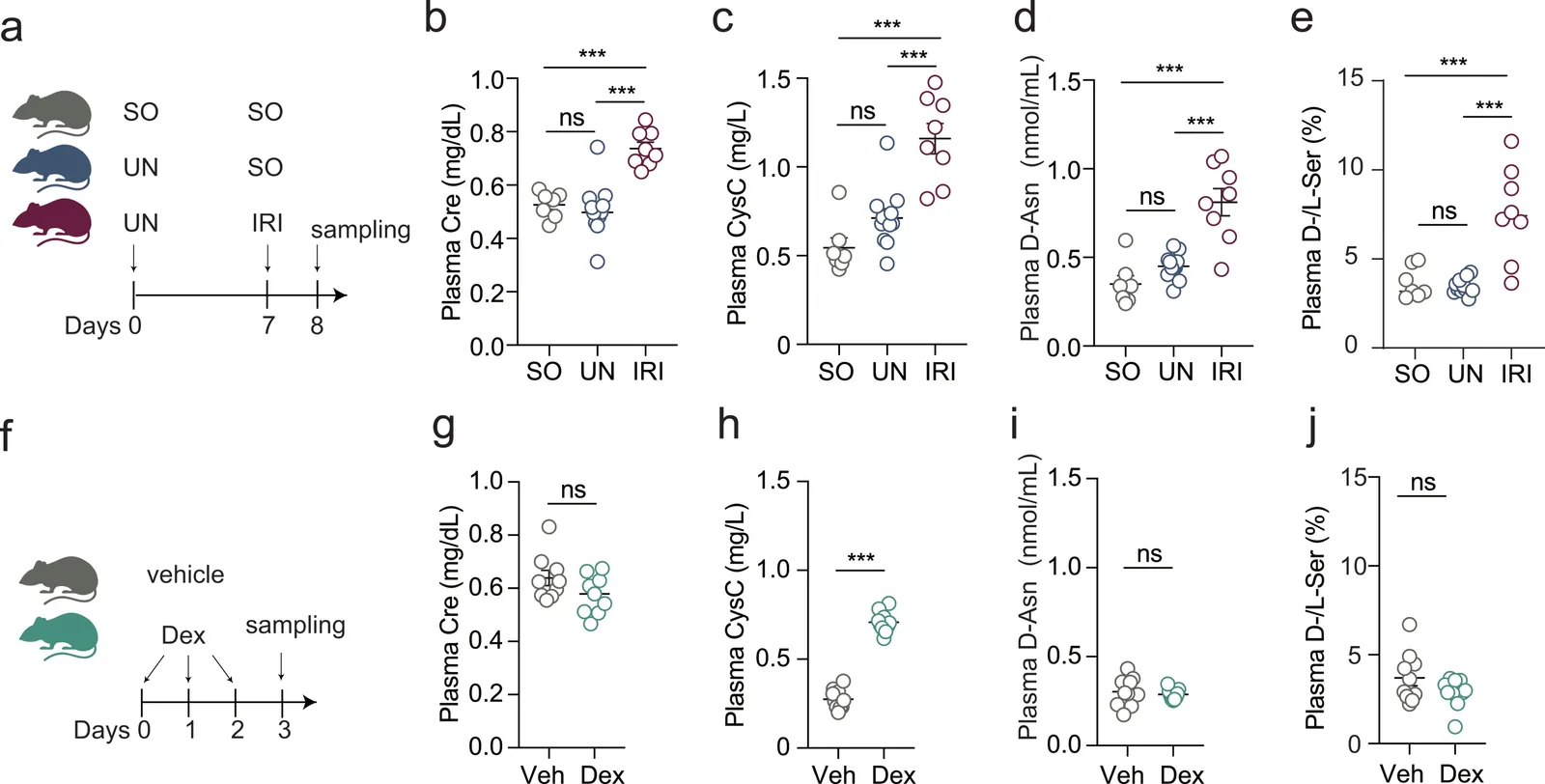
In mice, kidney dysfunction elevates plasma d-asparagine and the d/l-serine ratio, but dexamethasone does not. (**a**) Sham-operation (SO) twice for the SO group, UN/SO for the UN group, and UN/IRI for the IRI group was performed on C57BL6 mice. (**b-e**) Plasma Cre (b), CysC (c), d-asparagine (d), and d/l-serine ratio (e) in SO (*n* = 7), UN (*n* = 11), IRI mice (*n* = 8) were plotted. (**f**) C57BL6 mice were treated with Dex for three consecutive days. (**g-j**) Plasma Cre (g), CysC (h), d-asparagine (i), and d/l-serine ratio (**j**) in mice with vehicle (Veh, *n* = 10) and Dex (*n* = 10) treatment were plotted. Error bars, mean ± s.e.m. ***P < 0.001, analyzed with one-way ANOVA followed by Dunnett’s multiple comparisons test (**b**-**e**) or Mann Whitney U test (**g**-**j**). ‘ns’, not significant

Finally, we tested consequences of renal dysfunction on plasma d-asparagine and the d/l-serine ratio using developing C57BL6 mice. Renal dysfunction caused by renal ischemic reperfusion injury (IRI) after uninephrectomy (UN) significantly increased plasma d-asparagine as well as the d/l-serine ratio (with increased d- and decreased l-serine) compared to controls with sham operations. These were accompanied by elevation of serum creatinine and CysC (Fig. 5a-e and Supplementary Fig. S1de). In accordance with our findings of human CKD (Figs. 1 and 2), such increases of d-asparagine and the d/l-serine ratio had positive linear associations with serum creatinine and CysC (Supplementary Fig. 4ab). On the other hand, l-asparagine in the plasma was not altered by UN or IRI (Supplementary Fig. 4c). Urinary d/l-ratios of asparagine and serine had a tendency to decrease (Supplementary Fig. 4fg). These results suggest that renal damage affects aberrant d-amino acids.

Although serum CysC, one of the most reliable renal markers, is not significantly associated with body size or age in the juvenile population (Fig. 4a and Supplementary Fig. 3ef), CysC elevation by glucocorticoid administration is often a clinical problem in evaluating renal function. Therefore, we treated C57BL6 mice with dexamethasone (Dex) to test whether glucocorticoids impact plasma amino acid parameters (Fig. 5f). Consistent with previous studies (Risch et al. 2001; (Risch et al. 2005; Bokenkamp et al. 2007). CysC was significantly increased by Dex treatment (Fig. 5h). Dex treatment also mildly increased l-asparagine, but not l-serine with elevation of the liver enzyme ALT (Supplementary Fig. 5), which may be associated with enhanced asparagine synthesis under liver injury (Sun et al. 2023). On the other hand, Dex did not affect plasma d-asparagine, serine enantiomers, or creatinine (Fig. 5g-j and Supplementary Fig. 5b-e). Therefore, these results suggest that at least in mice, glucocorticoids do not influence d-asparagine or the d/l-serine ratio in plasma.

## Discussion

In this study, we found that plasma d-asparagine and the d/l-serine ratio reflect renal function and can accurately detect CKD in the pediatric population. In subjects with pediatric CKD and non-CKD, d-amino acids or d/l-amino acid ratios were clearly correlated with established renal markers and not with major non-renal biochemical parameters. Among enantiomers of asparagine and serine, FE of d-asparagine is as high as that of inulin, and plasma d-asparagine alone, or the d/l-serine ratio can detect pediatric CKD specifically and sensitively. Furthermore, d-asparagine and the d/l-serine ratio are not affected by body size, nor are they influenced by glucocorticoid administration in mice. Thus, plasma d-asparagine and the d/l-serine ratio can be clinically useful endogenous biomarkers in assessing renal function or detecting CKD during development or under steroid treatment.

Most amino acids detected in humans are their l-enantiomers. d-enantiomers are minor, but constantly produced by endogenous and exogenous (microbial) chiral-converting enzymes (Gonda et al. 2023; Sasabe and Suzuki 2019). Kidneys are critical in removing d-amino acids from circulation and maintaining dominance of l-enantiomers (Gonda et al. 2023). Therefore, kidney dysfunction has been associated with aberrant control of d-enantiomers in body fluids (Sasabe et al. 2014; Kimura et al. 2016). In middle-aged adult CKD, plasma d-serine and d-asparagine are negatively correlated with eGFR, and are linked to progression of CKD (Kimura et al. 2016). Such associations of d-serine and d-asparagine with renal function also hold true in the healthy adult population (Suzuki et al. 2022). Consonant with the study in adults, we confirmed that plasma d-asparagine and d-serine have clear linear associations with creatinine and CysC in children (Figs. 1 and 2).

Although plasma d-serine or d/l-serine ratio have clear correlations with kidney function (Figs. 1a and 2bd), these parameters had slight positive associations with age and body size in children (Fig. 4a), which may be linked to effects of sex hormones on expression of kidney d-serine transporter SMCTs (Wiriyasermkul et al. 2024; Hosoyamada et al. 2010). SMCTs also function cooperatively with urate transporter1 (URAT1) (Lu et al. 2013); therefore, they partly explain the correlation between d-serine and UA. In contrast, plasma d-asparagine is not affected by age or body size during development (Fig. 4). Unlike adults, pediatric physique changes significantly and rapidly, and renal biomarkers independent of physique or puberty are needed. Given the muscle mass-dependent production of creatinine, d-asparagine may be suitable for assessing renal function in children of different body sizes. Recently, Taniguchi et al. revealed that d-asparagine acts in a similar manner to inulin in adult kidneys (Taniguchi et al. 2023). Indeed, FE of d-asparagine in children is comparable to FE of inulin based on creatinine clearance in adults (Fig. 2f). Although larger-scale studies in children are needed, our findings support the idea that measurement of endogenous plasma d-asparagine is more practical than the invasive inulin test to evaluate renal function in children.

Pediatric CKD requires different management than adult CKD, since underlying diseases are mostly congenital and disease duration is longer than adult CKD. Therefore, early detection of CKD and accurate evaluation of renal function are critical for disease management. In children, however, eGFR based on creatinine, the most commonly measured endogenous filtration marker, requires correction by age, sex, race, and body size. This is because creatinine is affected not only by the level of GFR, but the level is also influenced by muscle mass, diet, and tubular secretion by active transport. Due to the absence of a universal equation to estimate GFR based on creatinine in children, evaluation of renal function as well as detection of CKD often relies on CysC. CysC is less affected by body size, but increases under steroid medication, which sometimes makes it difficult to accurately evaluate renal function. For instance, nephrotic syndrome, which requires steroid treatment, accompanies acute kidney injury at onset (Rheault et al. 2014; Sato et al. 2021). In addition to renal diseases, common diseases such as asthma and IgA vasculitis also require steroid therapy. Therefore, although there are many opportunities to examine patients with renal dysfunction medicated with steroids in pediatric practice, making it difficult in many cases to use existing biomarkers. In this study, we found that d-asparagine and/or the d/l-serine ratio can detect pediatric CKD sensitively and specifically without being affected by body size or steroids (Figs. 2 and 4, and 5). Although what confounds d-asparagine and/or the d/l-serine ratio remains to be determined, these parameters using d-amino acids would at least provide pediatricians with an option for assessing renal function when existing biomarkers are not clinically useful.

This study has the following limitations. First, we investigated Japanese children, an ethnically biased population. Second, due to the relatively low prevalence of CKD, sample size was limited. Third, since subjects with CKD were recruited from patients who had clinical follow-up for years, CKD subjects tended to be older and to have higher mass. Fourth, renal diseases included in this study are limited. Fifth, the control group was sex-biased, as females are more likely to be recruited due to menstruation and urinary tract infections. Sixth, morning urine collection is sometimes inaccurate before school age. Finally, since patients under steroid administration were excluded, the absence of any influence of steroids on d-asparagine found in mice may not be generalizable to humans.

The prevalence of pediatric CKD patients is increasing worldwide. Sensitive renal biomarkers independent of body size and medication with low invasiveness are needed to meet the need for early intervention. In this study, we show that plasma d-asparagine and the d/l-serine ratio correlate robustly with established renal biomarkers and can detect CKD sensitively and specifically. Since body size or steroid treatment do not influence plasma d-asparagine or the d/l-serine ratio, our findings provide useful options to evaluate renal function in children.

## Electronic supplementary material

Below is the link to the electronic supplementary material.

Supplementary Material 1

## Data Availability

No datasets were generated or analysed during the current study.
