# Supplementary material for: Plasma d-asparagine and the d/l-serine ratio reflect chronic kidney diseases in children regardless of physique
Source: Amino Acids. 2024 Jun 6;56(1):38. doi: 10.1007/s00726-024-03400-x (PMC11156734; doi:10.1007/s00726-024-03400-x)
Supplement: Supplementary file 1 — Supplementary Material 1 [file 726_2024_3400_MOESM1_ESM.pdf]

Supplementary Tables and Figures (Morishita et al.)
Supplementary Table 1. Characteristics of CKD group and control group

	**CKD (n=12)**	**Control (n=15)**	***P* value**
WBC	6500 ± 1902	6600 ± 1737	0.91
Neut, /μL	3771 ± 1416	3050 ± 1704	0.74
Lymp, /μL	1816 ± 1304	2361 ± 989	0.25
Hemoglobin, g/dL	14.3 ± 1.9	13.1 ± 0.7	0.13
Plt, $\times 10^4$ /μL	26.4 ± 6.4	31.1 ± 5.5	0.08
Albumin in the serum, g/dL	4.5 ± 0.3	4.4 ± 0.4	0.19
AST, IU/L	26.5 ± 9.7	30 ± 4.5	0.45
ALT, IU/L	16.5 ± 6.9	13 ± 3.7	0.022 *
BUN, mg/dL	19.5 ± 5.9	11 ± 2.3	0.001 **
UA, mg/dL	5.6 ± 1.0	3.7 ± 0.6	<0.0001 ****
Na, mEq/L	140 ± 1.4	140 ± 2.2	0.46
K, mEq/L	4.4 ± 0.4	4.2 ± 0.3	0.17
Cl, mEq/L	105 ± 1.7	104 ± 1.9	0.08
Ca, mg/dL	9.8 ± 0.3	9.8 ± 0.3	0.62
P, mg/dL	4.0 ± 0.95	4.9 ± 0.36	0.37
CRP, mg/dL	0.10 ± 0.26	0.075 ± 0.035	0.15
Glu, mg/dL	97 ± 9.9	90 ± 9.8	0.13
Urine specific gravity	1.019 ± 0.006	1.025 ± 0.007	0.013 *
Urinary protein / urinary creatinine, g/gCre	0.16 ± 0.38	0.04 ± 0.11	<0.0001 ****
Urinary calcium / urinary creatinine	0.05 ± 0.09	0.11 ± 0.06	0.025 *
FeNa, %	0.54 ± 0.31	0.38±0.14	0.025 *
FeK, %	6.3 ± 4.2	4.2 ± 3.3	0.17
%TRP, %	85.3 ± 4.5	93.1 ± 2.6	<0.0001 ****
NAG, U/L	5.2 ± 3.4	3.9 ± 2.5	0.085
Urinary β2-microglobulin / urinary creatinine, μg/mg	0.32 ± 4.38	0.11 ± 0.06	0.01 *

Supplementary Table 2. Characteristics of CAKUT and preterm group

	**CAKUT (n=7)**	**Preterm (n=5)**	***P* value**
Age, y	12.2 ± 5.6	15.2 ± 4.0	0.27
Male (%)	6 (86)	4 (80)	1
Height, cm	153.0 ± 32.5	145.2 ± 25.2	0.53
Body weight, kg	43 ± 20.9	53.7 ± 22.8	0.34
Body mass index, kg/m²	16.4 ± 4.04	20.5 ± 5.4	1
Body surface area, m^2	1.39 ± 0.47	1.47 ± 0.46	0.43
Gestational age, week	40 ± 0.93	29 ± 2.49	0.0043 **
Birth weight, g	3176 ± 155	930 ± 234	0.0043 **
Hypertension (%)	0 (0)	0 (0)	1
Hemoglobin, g/dL	12.8 ± 1.8	14.9 ± 0.7	0.028 *
Creatinine in the serum, mg/dL	0.83 ± 0.41	0.58 ± 0.24	0.64
Cystatin C in the serum, mg/dL	1.48 ± 0.43	1.02 ± 0.14	0.20
β2-miroglobulin in the serum, mg/dL	3.1 ± 1.0	1.6 ± 0.47	0.083
D-asparagine in the plasma, nmol/mL	0.28 ± 0.08	0.16 ± 0.05	0.011 *
L-asparagine in the plasma, nmol/mL	52.4 ± 8.3	44.3 ± 8.9	1
D-/L-asparagine ratio in the plasma, %	0.49 ± 0.11	0.37 ± 0.06	0.073
D-serine in the plasma, nmol/mL	3.08 ± 1.10	1.91 ± 0.60	0.11
L-serine in the plasma, nmol/mL	111 ± 16.0	117 ± 13.2	0.97
D-/L-serine ratio in the plasma, %	2.77 ± 0.69	1.62 ± 0.37	0.048 *
Urinary D-asparagine / urinary creatinine, nmol/mg	24.3 ± 12.5	23.2 ± 14.2	0.64
Urinary L-asparagine / urinary creatinine, nmol/mg	109 ± 51	73.3 ± 59.7	0.76
Urinary D-serine / urinary creatinine, nmol/mg	139.6 ± 59.6	138.2 ± 30.1	0.34
Urinary L-serine / urinary creatinine, nmol/mg	190.0 ± 154.4	151.4 ± 126.8	0.53
D-/L-asparagine ratio in the urine, %	56.8 ± 25.7	81.3 ± 40.4	0.76
D-/L-serine ratio in the urine, %	103.3 ± 92.7	111.0 ± 39.3	0.76
Creatinine based $FE_{D\text{-asparagine}}$, %	68.7 ± 16.5	65.7 ± 35.0	0.88
Creatinine based FED-serine, %	45.1 ± 11.3	53.0 ± 11.3	0.76
eGFR_Cre, mL/min/1.73 m²	56.8 ± 16.6	77.5 ± 16.1	0.15
eGFR_CysC, mL/min/1.73 m²	62.5 ± 23.7	87.7 ± 10.1	0.27
eGFR_β2-microglobulin, mL/min/1.73 m²	57.2 ± 21.6	102.3 ± 20.4	0.083

Supplementary Figure 1.

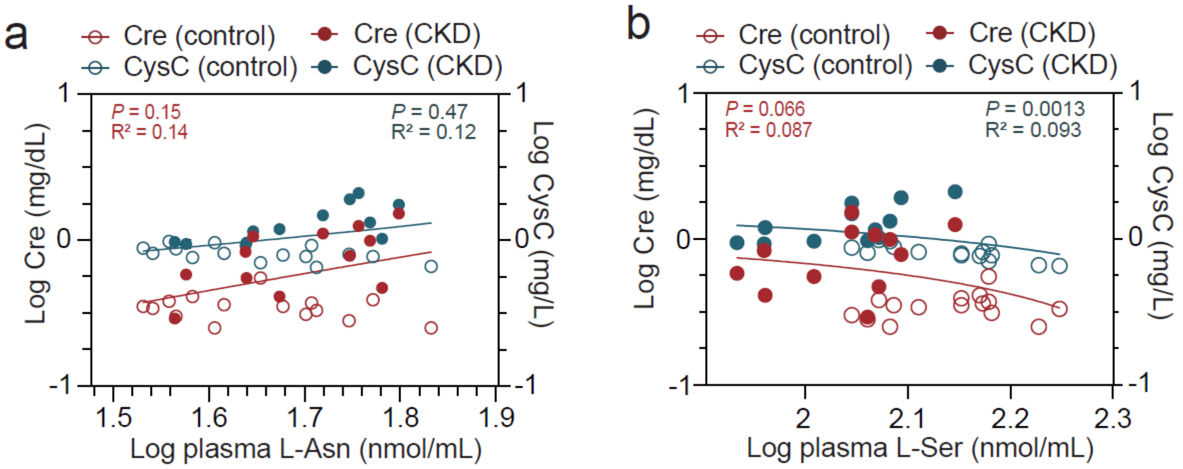

Correlations of plasma L-amino acids with kidney parameters in children with CKD and controls. (**a, b**) Linear regression shows associations of plasma L-asparagine (a) or L-serine (b) with serum Cre and CysC in children with CKD (closed circles, n = 12) and controls (open circles, n = 15).

Supplementary Figure 2.

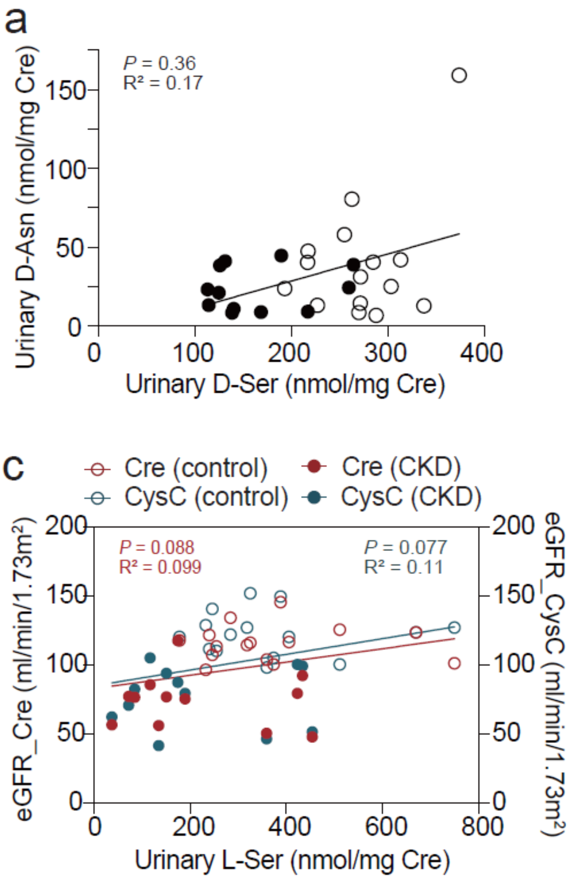

Correlations of urinary amino acid enantiomers with kidney parameters in children with CKD and controls. (**a-d**) Linear regression indicates associations between urinary D-serine and D-asparagine (a); urinary L-serine and L-asparagine (b); urinary L-serine and eGFR_Cre/CysC (c); or urinary L-asparagine and eGFR_Cre/CysC (d) in children with CKD (closed circles, n = 12) and controls (open circles, n = 15).

Supplementary Figure 3.

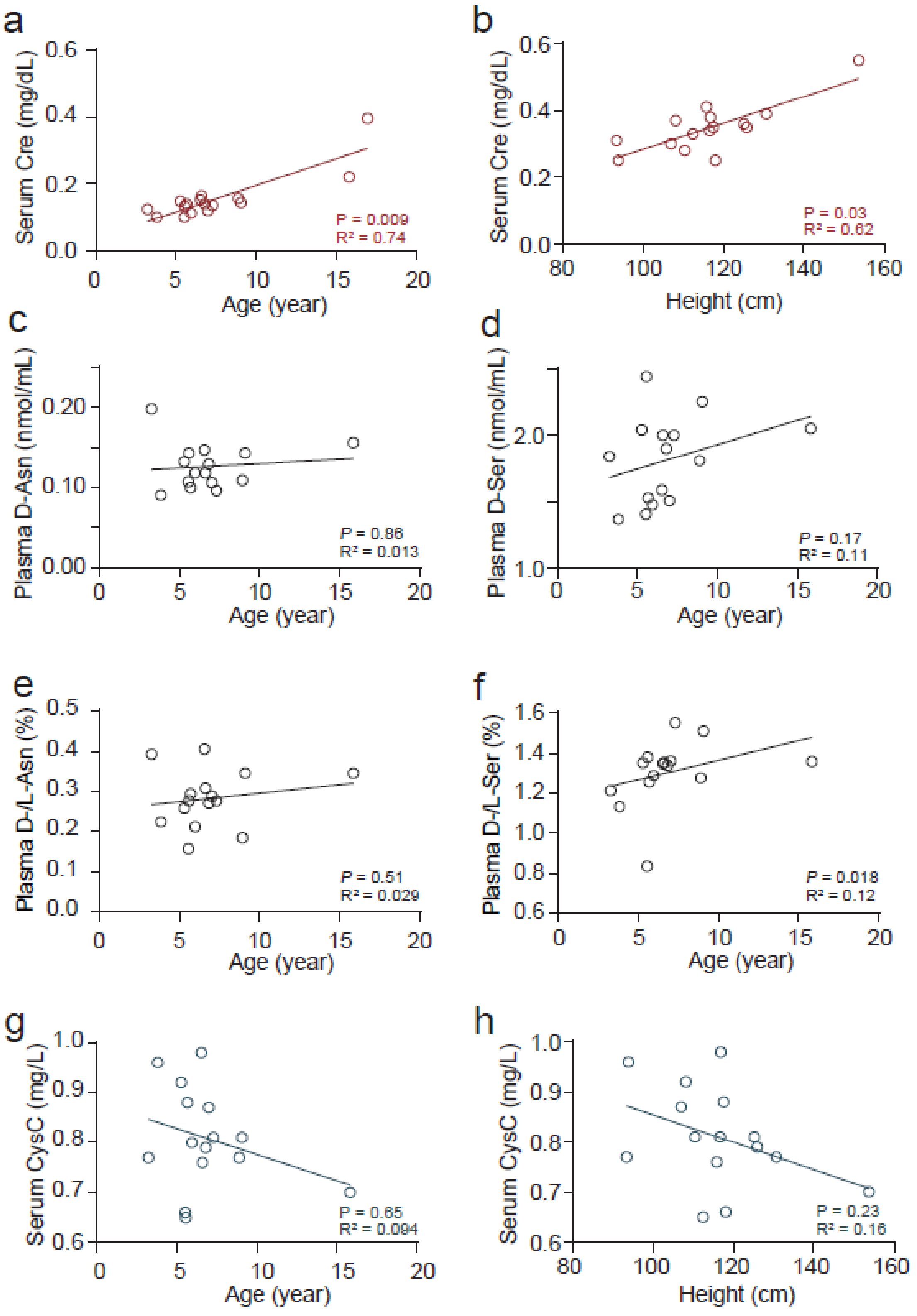

Correlations of kidney parameters with age/height in non-CKD children. (**a-h**) Linear regression shows associations between serum Cre and age (a); serum Cre and height (b); plasma D-asparagine and age (c); plasma D-serine and age (d); plasma D-/L-asparagine ratio and age (e); plasma D-/L-serine ratio and age (f); serum CysC and age (g); or serum CysC and height (h) in non-CKD control children (n = 15).

Supplementary Figure 4.

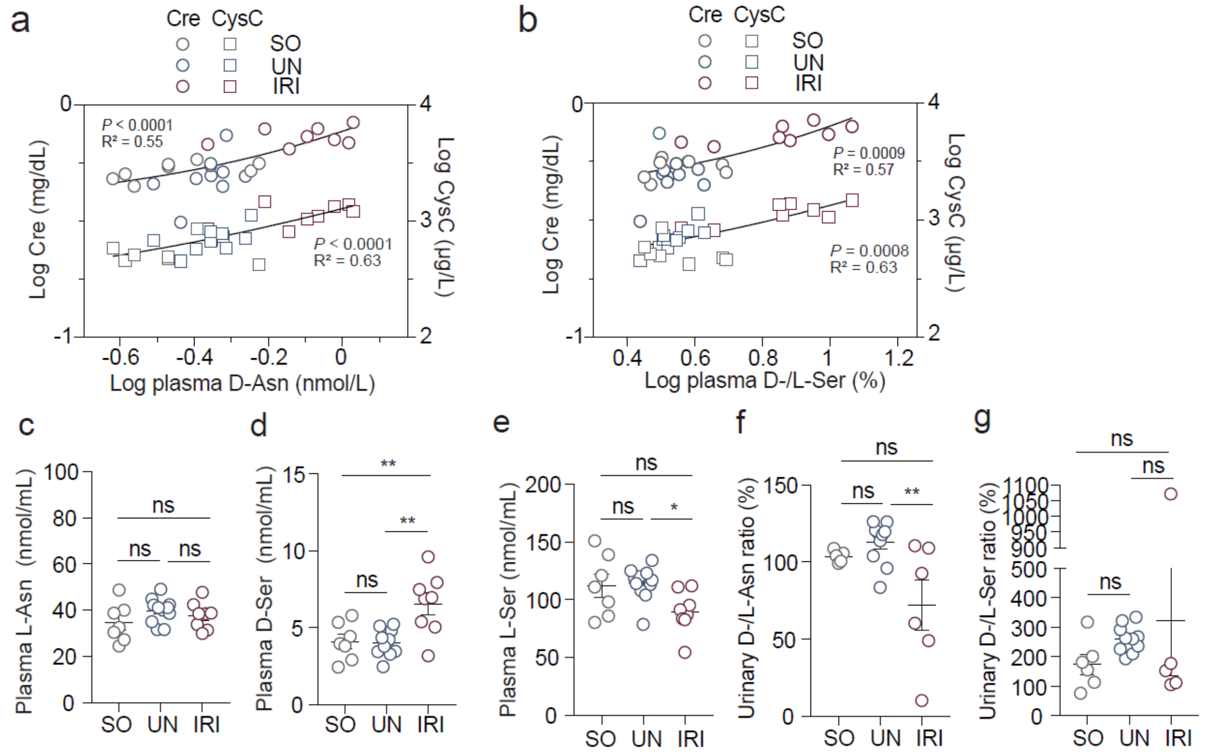

Plasma D-asparagine and the D-/L-serine ratio correlate with plasma Cre and CysC in mice with kidney dysfunction. (**a, b**) Linear regression show associations between plasma D-asparagine and Cre/CysC (a); or the plasma D-/L-serine ratio and Cre/CysC (b) in SO (n = 7), UN (n = 11), and IRI mice (n = 8). (**c-g**) Plasma D-asparagine (c), plasma D-serine (d), plasma L-serine (e), the urinary D-/L-asparagine ratio (f), and the urinary D-/L-serine ratio in SO (n = 7), UN (n = 11), and IRI mice (n = 8) are plotted. Error bars, mean ± s.e.m. **P* < 0.05, ***P* < 0.01, ****P* < 0.001, analyzed with one-way ANOVA followed by Dunnett's multiple comparisons test. 'ns', not significant.

Supplementary Figure 5.

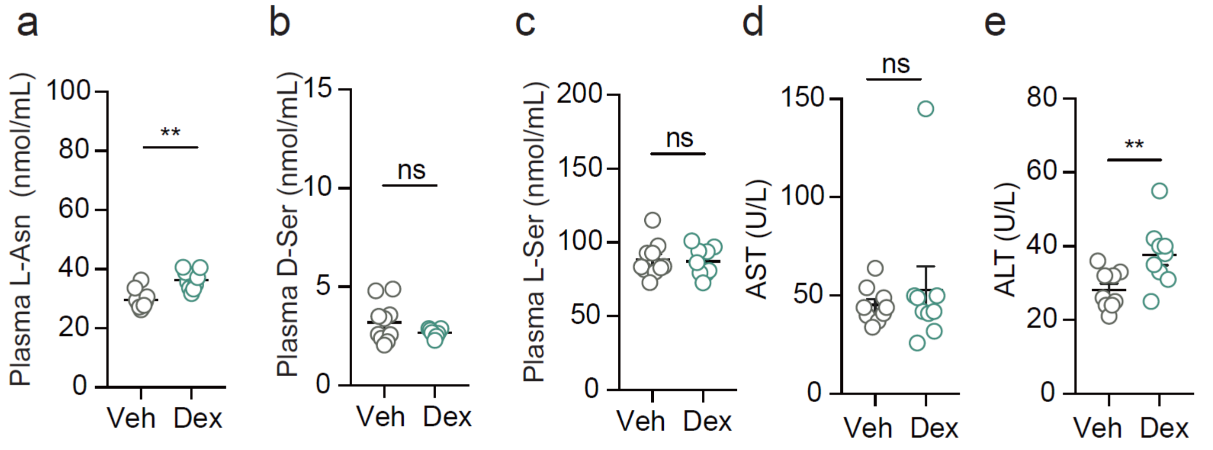

Effect of Dex treatment on amino acid enantiomers in mice. (**a-e**) Plasma L-asparagine (a), D-serine (b), L-serine (c), AST (d), and ALT in SO (n = 7), UN (n = 11), and IRI mice (n = 8) are plotted. Error bars, mean ± s.e.m. $^{**}P < 0.01$, analyzed with Mann Whitney U test. 'ns', not significant.
